# Zebrafish VEGF Receptors: A Guideline to Nomenclature

**DOI:** 10.1371/journal.pgen.1000064

**Published:** 2008-05-30

**Authors:** Jeroen Bussmann, Nathan Lawson, Leonard Zon, Stefan Schulte-Merker, Zebrafish Nomenclature Committee

**Affiliations:** 1 Hubrecht Laboratory (NIOB-KNAW NIOB), Utrecht, The Netherlands; 2 University of Massachusetts Medical School, Worcester, Massachusetts, United States of America; 3 Harvard Medical School and Children's Hospital Pediatrics, Cambridge, Massachusetts, United States of America; 4 Hubrecht Laboratory (NIOB-KNAW NIOB), Utrecht, The Netherlands; 5 Marc Ekker, Center for Advanced Research in Environmental Genomics, University of Ottawa, Ontario, Canada; Mary Mullins, Department of Cell and Developmental Biology, University of Pennsylvania, Philadelphia, Pennsylvania, United States of America; John Postlethwait, Institute of Neuroscience, University of Oregon, Eugene, Oregon, United States of America; Monte Westerfield, Institute of Neuroscience, University of Oregon, Eugene, Oregon, United States of America; The Jackson Laboratory, United States of America

## Introduction

In placental mammals (eutherians), there exist three paralogous genes of the vascular endothelial growth factor (VEGF) receptor family, namely FLT1 (also named VEGFR1), KDR (also named FLK1 and VEGFR2), and FLT4 (also named VEGFR3). Recent analysis of the VEGF receptor repertoire in basally diverging vertebrates has identified a fourth representative of this gene family, which was secondarily lost within the eutherian lineage, but is still present in marsupials and platypus (monotremata). Because this fourth member was initially described as an orthologue of the human KDR gene in zebrafish, confusion has arisen regarding the evolutionary relationships of vertebrate VEGF receptors. Here, we revise the nomenclature of zebrafish VEGF receptors and name the fourth vertebrate VEGF receptor gene *kdr-like*.

The members of the VEGF family of ligands, among them VEGF-A, VEGF-B, and VEGF-C, mediate cellular responses by binding their cognate receptors. The receptors, which belong to the type III receptor tyrosine kinase family, are single-pass transmembrane proteins containing seven extracellular immunoglobulin domains and a split intracellular tyrosine kinase domain. In human, mouse, and other mammals, three VEGF receptors have been identified, namely FLT1 (also named VEGFR1), KDR (also named FLK1 and VEGFR2), and FLT4 (also named VEGFR3).

Since their initial identification in mammals, proteins homologous to VEGFRs have been identified in several basally diverging vertebrates, including birds [1],[2], amphibians [3], and teleost fish. In the zebrafish, four genes encoding VEGF receptor proteins have been identified: the FLT1 orthologue [4],[5], the FLT4 orthologue [6], and two genes with highest similarity to KDR/Flk1. The first of these to be cloned [6]–[9] and functionally characterized [10] has been used in more than 80 papers as a marker of endothelial cells in the zebrafish and was originally named as the zebrafish orthologue of KDR/FLK1. However, the recent identification of a second potential KDR/FLK1 orthologue [4],[11],[12], which in fact is more similar to the human gene, has caused confusion over the evolutionary relationships of zebrafish and mammalian VEGF receptors.

**Figure 1:**
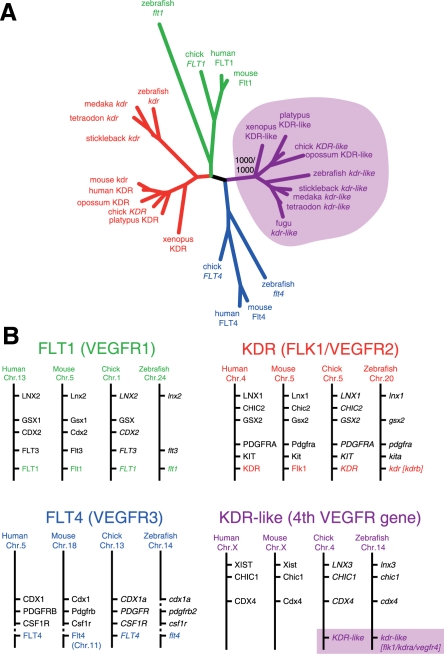
Adapted from Bussmann et al., 2007 [4]. A. Unrooted neighbor-joining tree of vertebrate VEGF receptors. Different colors represent different classes of VEGF receptors. Purple shading was used to highlight the fourth vertebrate VEGF receptor class, which is missing in eutherian mammals. The node representing this fourth class was supported in 1000/1000 bootstrap replicates. B. Synteny analysis of vertebrate VEGF receptors in the human, mouse, chick, and zebrafish genome assemblies. Different classes in A and B are colored similarly. Dashed lines represent synteny breaks in the Flt4 loci. Brackets indicate previously suggested names.

In many cases, the presence of two zebrafish orthologues of a single human gene can be attributed to a whole-genome duplication event that occurred within the teleost lineage. It was therefore hypothesized that zebrafish contains duplicated KDR genes, which were consequently called *kdra* (the gene originally called *flk1*) and *kdrb* (the gene that is most similar to human KDR). However, two lines of evidence have recently challenged this view [4], [13]–[15] and rather suggest that this is a case of “ohnologs” [16],[17]. First, VEGF receptor sequences that were most similar to zebrafish *flk1/kdra* were identified, not only in the genomes of other teleosts, but also in the genomes of higher vertebrates, such as Xenopus, chicken, platypus, and opossum. Phylogenetic analysis of these genes, together with other VEGF receptor sequences, clearly showed that they cluster as a separate class (Figure 1A). Second, synteny analysis showed that the loci containing the zebrafish *flk1/kdra* and *kdrb* have been conserved throughout vertebrate evolution (Figure 1B), strongly indicating the presence of both genes in the common ancestor of fish and mammals and the loss of a fourth VEGF receptor in the eutherian lineage (after the divergence of marsupial and placental mammals).

Although representing separate classes, experimental data revealed significant functional similarity of zebrafish *flk1/kdra* and *kdrb.* Both genes are expressed in all endothelial cells, whereas *flt1* and *flt4* have a more restricted expression. Furthermore, zebrafish VEGF can bind and activate both *flk1/kdra* and *kdrb* [11]. Finally, *flk1/kdra* and *kdrb* genetically interact: knockdown of *kdrb* in a *flk1/kdra* mutant background resulted in similar phenotypes as those observed in embryos in which *vegf* was knocked down or in which a downstream signaling component, *phospholipase-cγ1*, is mutated ([12] and N. Lawson, unpublished data).

## Conclusion

Therefore, to reflect that *flk1/kdra* is a prominent receptor in VEGF-A signaling in zebrafish, while at the same time indicating that it represents a fourth class of vertebrate VEGF receptors (and is not the result of a teleost gene duplication), we propose to rename this gene *kdr-like*. As the zebrafish *kdrb* gene is clearly orthologous to mammalian KDR, we propose to rename this gene *kdr.*
